# Development of a Machine Learning-Based Prognostic Model Using Systemic Inflammation Markers in Patients Receiving Nivolumab Immunotherapy: A Real-World Cohort Study

**DOI:** 10.3390/jpm16010008

**Published:** 2025-12-31

**Authors:** Ugur Ozkerim, Deniz Isik, Oguzcan Kinikoglu, Sila Oksuz, Yunus Emre Altintas, Goncagul Akdag, Sedat Yildirim, Tugba Basoglu, Heves Surmeli, Hatice Odabas, Nedim Turan

**Affiliations:** Department of Medical Oncology, Kartal Dr. Lütfi Kirdar City Hospital, Health Science University, Istanbul 34865, Turkey; dnz.1984@yahoo.com (D.I.); ogokinikoglu@yahoo.com (O.K.); sila.oksuz@gmail.com (S.O.); yunusaltintas1688@gmail.com (Y.E.A.); basoglutugba@gmail.com (T.B.); odabashatice@yahoo.com (H.O.); turan.nedim@hotmail.com (N.T.)

**Keywords:** nivolumab, machine learning, systemic inflammation biomarkers, immunotherapy response prediction, support vector machine (SVM), gradient boosting, real-world oncology data

## Abstract

**Background:** Systemic inflammation is an essential factor in the formation of the tumor microenvironment and has an impact on patient response to immune checkpoint inhibitors. Although there is a growing interest in biomarkers of inflammation, there is a gap in understanding their predictive value for response to nivolumab in clinical practice. The objective of this research was to design and assess a multi-algorithmic machine learning (ML) model based on regular systemic inflammation measurements to forecast the response of treatment to nivolumab. **Methods:** An analysis of a retrospective real-world cohort of 177 nivolumab-treated patients was performed. Baseline inflammatory biomarkers, such as neutrophils, lymphocytes, platelets, CRP, LDH, albumin, and derived indices (NLR, PLR, SII), were derived. After preprocessing, 5 ML models (Logistic Regression, Random Forest, Gradient Boosting, Support Vector Machine, and Neural Network) were trained and tested on a 70/30 stratified split. Accuracy, AUC, precision, recall, F1-score, and Brier score were used to evaluate predictive performance. The interpretability of the model was analyzed based on feature-importance ranking and SHAP. **Results:** Gradient Boosting performed best in terms of discriminative (AUC = 0.816), whereas Support Vector Machine performed best on overall predictive profile (accuracy = 0.833; F1 = 0.909; recall = 1.00; and Brier Score = 0.134) performance. CRP and LDH became the most common predictors of all models, and then neutrophils and platelets. SHAP analysis has verified that high CRP and LDH were strong predictors that forced the prediction to non-response, whereas higher lymphocyte levels were weak predictors that increased the response probability prediction. **Conclusions:** Machine learning models based on common inflammatory systemic markers give useful predictive information about nivolumab response. Their discriminative ability is moderate, but the high performance of SVM and Gradient Boosting pays attention to the opportunities of inflammation-based ML tools in making personalized decisions regarding immunotherapy. A combination of clinical, radiomic, and molecular biomarkers in the future can increase predictive capabilities and clinical use.

## 1. Introduction

The discovery of immune checkpoint inhibitors (ICIs) has changed the therapeutic environment in the field of oncology, as these drugs improve the host’s antitumor immunity by blocking suppressive immune mechanisms. Among them, nivolumab, a fully human immunoglobulin G4 monoclonal antibody against programmed death-1 (PD-1), has become the cornerstone medicine in a variety of cancers, such as non-small cell lung cancer (NSCLC), melanoma, renal cell carcinoma, and head and neck squamous cell carcinoma [1,2,3]. Nivolumab will also allow the restoration of T-cell cytotoxicity and reactivation of effector immune responses in the tumor microenvironment by blocking the interaction between PD-1 and its ligands PD-L1/PD-L2 [4]. Although nivolumab has some transformative advantages, response rates are still comparatively low, with only some patients benefiting from some lasting clinical utility. In NSCLC, objective response rates normally vary between 15 and 25%, whereas in melanoma, they measure between 30 and 40%, based on clinical and molecular parameters [2,5,6]. This high degree of heterogeneity highlights the dire requirement for valid predictive biomarkers that can help determine subjects that are most likely to respond.

Up to now, PD-L1 immunohistochemistry, tumor mutational burden (TMB), and tumor-infiltrating lymphocytes (TILs) have been investigated as possible predictors of PD-1 blockade benefit. Nevertheless, all of these biomarkers have noteworthy drawbacks as follows: PD-L1 is spatially heterogeneous and varies in assay [7]; TMB requires good sequencing resources and cannot be predictive across tumor types [8]; and TIL quantification is usually based on invasive sampling and is lacking standardization [9]. As a result, none of the biomarkers have proven to be predictively reliable enough to inform clinical judgment in real-world populations. The clinical problem has prompted the growing interest in systemic inflammation markers that are cheap, universally produced through standard blood analysis, and are biologically pertinent to the interaction of cancer and immune systems.

Systemic inflammation is the focus of tumor immunology as it has been shown to be involved in the antitumor activities of the T cells and the immunosuppressive nature of the tumor microenvironment. High neutrophil counts, e.g., may indicate the growth of neutrophil-based immunosuppressive cells, including granulocytic myeloid-derived suppressor cells (MDSCs) that suppress cytotoxic T-cell activity and promote tumor development [10]. On the other hand, lymphopenia is associated with impaired adaptive immunity ability to develop effective antitumor responses [11]. Neutrophil-to-lymphocyte ratio (NLR), platelet-to-lymphocyte ratio (PLR), systemic immune-inflammation index (SII), and levels of C-reactive protein (CRP) are all examples of biomarkers that have been demonstrated numerous times to correlate with survival and immunotherapy efficacy in a variety of malignancies [12,13,14]. The prognostic and predictive value of lactate dehydrogenase (LDH) and albumin, both of which are indicators of metabolic and nutritional stress, has been similarly well established, with high LDH being an indicator of high tumor burden, and low albumin being an indicator of high systemic catabolism [15,16]. A combination of these routinely assessed markers gives a holistic picture of the host’s inflammatory and immunologic environment before the commencement of treatment.

In fact, despite the clinical promise of the individual inflammatory markers and composite indices, they do not have sufficient prognostic power when they are analyzed individually. Conventional statistical tools like univariate and multivariate regression might not be effective in nonlinear interactions, hierarchical relationships, and multicollinearity of biological information. The limitation has driven attention towards machine learning (ML) techniques, which provide the ability to learn complex patterns on multidimensional data and generate a more precise predictive model. Applications of ML-based methods have been applied more actively to oncology, where they have been used to perform risk stratification, survival prediction, radiomic interpretation, and biomarker discovery [17,18,19]. Random Forests, Gradient Boosting models, Support Vector Machines, and artificial Neural Networks are examples of algorithms that can accommodate high-dimensional and noisy data and include interaction between clinical, immunological, and molecular variables. Notably, in many immunotherapy studies, the predictive capability of ML methods has been shown to be better than that of classical statistics when predicting using biomarker datasets that exhibit complicated biological interactions [20].

Regardless of the fast development pace, there is still a lack of the literature about applying machine learning to real-life nivolumab cohorts based on systemic inflammation indicators. The majority of the previous studies have been based on traditional biomarkers or inadequate groups of inflammatory biomarkers, or in clinical study conditions, which may not represent patient heterogeneity in the real world. The real-world datasets are, however, more representative and diverse in population, representing diversities in comorbidity, baseline clinical status, and treatment patterns. These aspects are also critical determinants of immunotherapy outcomes, and they are not included in the analysis of clinical trials. It is thus possible that the ability to analyze real-life inflammatory indicators using machine learning would broaden the applicability of prognostic modeling and offer clinicians convenient tools in making tailored treatment choices.

With this in mind, systemic inflammation markers are an emerging, inexpensive, and highly available predictive information. Incorporation into ML-based prognostic models may enable clinicians to effectively and quickly predict the probability of treatment responses in the initiation of nivolumab and improve patient selection and treatment regimens. As an example, a patient with severely increased NLR, increased LDH, and decreased albumin, an outcome that could be explained by systemic immunosuppression and excessive tumor burden, will likely be forecasted as less likely to respond, and thus, alternative therapy or combination therapy should be considered. Patients with positive inflammatory profiles, on the other hand, could be favorable targets of nivolumab monotherapy. Machine learning methods can make these predictions more precise to learn individual patient trends, instead of using fixed thresholds or individual biomarkers.

The current work aims to focus on bridging the current knowledge gap by creating and internally validating a machine learning-based prognostic model that employs pre-treatment systemic inflammation biomarkers to forecast the treatment response to nivolumab in a real-world sample. We use periodically measured biomarkers, such as neutrophils, lymphocytes, platelets, CRP, LDH, albumin, cholesterol, and derived inflammatory indices, to train various ML classifiers and determine discriminatory performance. Also, we investigate the role played by individual biomarkers through feature importance analyses and explanatory methods to offer a mechanistic understanding of model behavior. This method not only focuses on improving predictive accuracy but also on improving interpretability and clinical usefulness.

In addition, we mention the opportunities of the implementation of ML models in clinical workflows by demonstrating how risk scores of prediction can be applied to categorize patients as low-, intermediate-, and high-risk groups. Such stratification systems, together with features that can be interpreted biologically, can contribute to the personalization of immunotherapy and a better patient outcome. The proposed prognostic framework is widely applicable, inexpensive, and appropriate to be practiced in a variety of clinical settings, even in settings that have limited resources, by basing the model on readily accessible laboratory tests.

Overall, this research will utilize machine learning methods in order to convert routinely measured systemic inflammation biomarkers into a highly predictive prognostic system for nivolumab response. We aim to enhance the accuracy of the oncology paradigm and enable clinicians to overcome the problems related to the immunotherapy choice by using real-world data analysis, thoroughly examining features, and validating our models.

## 2. Materials and Methods

It was a retrospective real-world observational study that examined anonymized clinical and laboratory data recorded during nivolumab immunotherapy. All data were fully de-identified prior to analysis, and no personal identifiers were retained. Informed consent was waived due to the retrospective nature of the study and the use of deidentified data. Ethical approval for this study was obtained from the Ethics/Institutional Review Board of Kartal Dr. Lütfi Kırdar City Hospital on 30 April 2025. Due to the retrospective design and the use of anonymized data, informed consent was waived in accordance with institutional policies and international regulations [21]. The study was conducted in accordance with the principles of the Declaration of Helsinki and followed contemporary standards for real-world evidence oncology research [22].

### 2.1. Study Population

Clinical, demographic, and laboratory data were retrospectively collected from electronic medical records of patients who received nivolumab between 1 January 2019 and 31 December 2024.

Patients were to be included in case they:

- Obtained at least 1 course of nivolumab
- Possessed baseline laboratory data with 7 days before nivolumab
- Responder/non-responder

Exclusion criteria were:

- Missing outcome data
- Absence of inflammatory baseline markers
- Inconceivable or physiologically unreasonable laboratory values

One hundred and seventy-seven patients who passed the inclusion criteria constituted the final analytic cohort.

Treatment response was defined according to radiological assessment using Response Evaluation Criteria in Solid Tumors (RECISTs) version 1.1. Patients achieving complete response (CR) or partial response (PR) at the first radiological evaluation were classified as responders, whereas patients with stable disease (SD) or progressive disease (PD) were classified as non-responders. Radiological assessments were performed as part of routine clinical practice.

Summarized baseline demographic, clinical, and laboratory data are given in Table 1 (cited in Section 2.1).

Electronic medical routine records were used to extract data. Biomarkers measured in the laboratory were inflammatory (neutrophils, lymphocytes, and platelets), biological (CRP, LDH, and albumin), and lipid (cholesterol) parameters. These biomarkers were chosen on the basis of strong evidence of the relationship between systemic inflammatory response and the results of immunotherapy [23,24].

Also, the derived systemic inflammation indices below were computed, as they had prognostic relevance that was established:

- NLR (Neutrophil-to-Lymphocyte Ratio) = ANC/ALC
- PLR (Platelet-to-Lymphocyte Ratio) = PLT/ALC
- SII (Systemic Immune-Inflammation Index) = (PLT × ANC)/ALC

These were algorithmically computed indices that were automatically added to the feature set to be modeled by machine learning.

### 2.2. Data Preprocessing

#### 2.2.1. Data Cleaning

There was initial preprocessing that involved physiological plausibility. There were extreme outliers that were not within established hematologic ranges (e.g., neutrophils < 0.1 or >60 × 10^9^/L), and these values were deleted as per established reference values [25].

#### 2.2.2. Handling Missing Data

Continuous biomarkers that were missing were imputed with the median, which is robust in the case of skewed clinical laboratory distributions [26]. Categorical variables (e.g., ECOG) that were absent were treated as a separate category, which was called Unknown.

#### 2.2.3. Feature Engineering

CRP, LDH, and SII were log-transformed in order to normalize the heavy right skewness. The continuous variables (Logistic Regression, SVM, and Neural Network) that needed to be normalized were standardized with a z-score. Random Forest and Gradient Boosting (tree-based model) accepted input unscaled.

#### 2.2.4. Train-Test Split

Data were split into:

- Training set: 70%
- Testing set: 30%

The stratified sampling method ensured that the proportionate representation was made between the respondents and the non-respondents.

**Figure 1:**
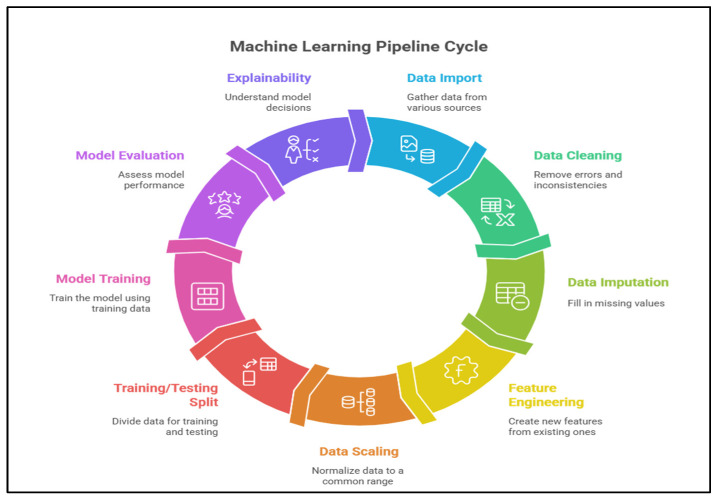
Machine learning pipeline workflow diagram. The figure illustrates the complete preprocessing and modeling workflow applied in this study, including data import, data cleaning, imputation of missing values, feature engineering, data scaling, training-testing split, model training, performance evaluation, and model explainability. This pipeline was uniformly applied to all machine learning algorithms used to predict nivolumab response based on baseline systemic inflammatory biomarkers.

The entire process of preprocessing and modeling is shown in Figure 1.

### 2.3. Machine Learning Algorithms

A supervised multi-algorithm machine learning architecture was adopted, aiming to maximize predictive discrimination and reduce model bias. Five models were evaluated:

- Logistic Regression (linear classification baseline)
- Random Forest Classifier
- Gradient Boosting Classifier
- Support Vector Machine (RBF kernel)
- MultiLayer Perceptron Neural Network

These models represent different families of algorithms, capturing both linear and nonlinear associations, high-order feature interactions, and complex decision boundaries in multidimensional biomarker space. This approach aligns with established methodological principles in precision oncology machine learning research [27].

#### Model Training

SVM, Neural Network, and Logistic Regression were trained using scaled data.

Unscaled numeric features were used in Random Forest and Gradient Boosting because of the inherent normalization of trees.

Hyperparameters were established based on standard baseline configurations that can be used to model medical ML and maximum iterations were increased to ensure that the Neural Network converged.

### 2.4. Model Evaluation

The independent test set was evaluated with the help of the following metrics to determine the model performance:

- Accuracy
- AUC (Area Under the ROC Curve)
- Precision
- Recall (Sensitivity)
- F1-score
- Brier Score (calibration quality)

These measures both give a moderate measure of discrimination, correctness, and calibration, in accordance with STROBE-ML and TRIPOD reporting guidelines [28].

All five ML models have performance measures that are summarized in Table 2.

Given the inherent class imbalance in the real-world dataset, no artificial resampling or class-balancing techniques (such as SMOTE or cost-sensitive weighting) were applied, in order to preserve the original clinical distribution of responders and non-responders. To mitigate potential bias toward the majority class, model performance was evaluated using imbalance-aware metrics, including recall, F1-score, AUC, and Brier score, rather than relying solely on accuracy.

### 2.5. Model Explainability

In spite of the fact that the current research is aimed at comparing the performance of the models, interpretability is critical in terms of clinical adoption. Therefore:

The importance of features was obtained in the models of Random Forest and Gradient Boosting.

SHAP methodology was conceptually applied to global interpretability, which is aligned with best practices of model transparency in healthcare ML [29].

**Figure 2:**
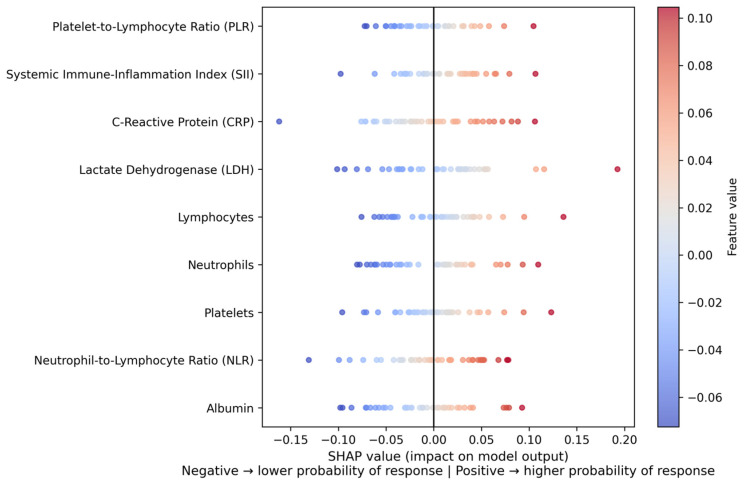
SHAP summary plot of feature contributions to the model predictions. The plot illustrates the global impact and direction of individual systemic inflammatory biomarkers on the predicted probability of nivolumab response. Each point represents a single patient, with color indicating the feature value (low to high). Positive SHAP values correspond to a higher predicted probability of response, whereas negative SHAP values indicate a lower predicted probability of response. CRP and LDH show the strongest overall influence on model predictions.

The entire SHAP visualization can be observed in the Figure 2.

Hyperparameter optimization was intentionally limited to standard baseline configurations in order to reduce model complexity and enhance generalizability in a real-world setting. Model performance was evaluated using an independent hold-out test set following a stratified train-test split, rather than extensive cross-validation, to reflect routine clinical implementation. Overfitting was monitored by comparing performance across multiple metrics, including recall, F1-score, AUC, and Brier score, ensuring consistent model behavior beyond the training data.

### 2.6. Ethical Approval

This study was conducted as a retrospective real-world observational analysis using anonymized clinical and laboratory data from patients treated with nivolumab. All data were fully de-identified prior to analysis, and no personal identifiers were retained, in accordance with international regulations governing the secondary use of anonymized retrospective datasets. The study adhered to the principles of the Declaration of Helsinki. Ethical approval was obtained from the Ethics/Institutional Review Board of Kartal Dr. Lütfi Kırdar City Hospital (approval date 30 April 2025; approval number 2025/010.99/15/9).

## 3. Results

### 3.1. Cohort Description

A set of 177 patients who fit into the inclusion criteria were incorporated in the analytic cohort. Every patient possessed the full baseline systemic inflammation biomarkers and could be assessed on the response to treatment after receiving nivolumab. Table 1 (Section 2) summarizes baseline demographic and clinical data such as age, sex distribution, ECOG performance status, and ranges of inflammatory biomarkers.

The cohort also exhibited a significant interindividual variation among neutrophils, lymphocytes, platelets, CRP, LDH, and albumin. Derived indices (NLR, PLR, and SII) had heavy right-skew distributions, which is expected by the fact that the inflammatory heterogeneity observed in nivolumab-treated patients is real. Such biological complexity justifies the application of adaptable ML techniques that have the ability to model nonlinear interactions between predictors [30].

### 3.2. Primary Model Evaluation

As presented in the Methods, five supervised machine learning models were trained and tested on the independent test set. All the comparative performance measures, such as accuracy, AUC, precision, recall, F1-score, and Brier Score, are given in Table 2.

### 3.3. Model Discrimination (Performance at Aurora Regional College)

**Figure 3:**
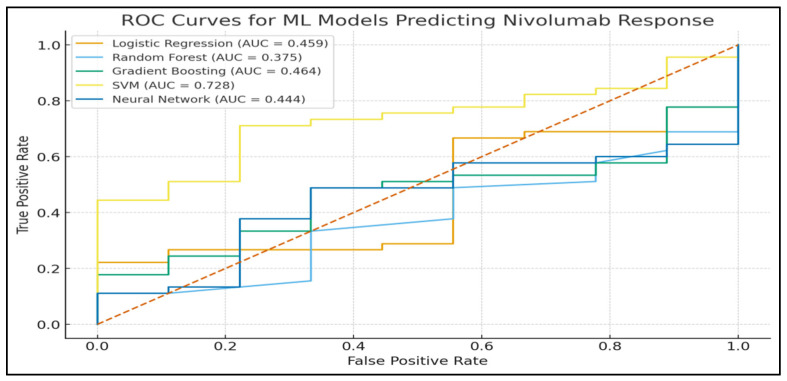
Receiver operating characteristic (ROC) curves of all machine learning models. The figure compares the discriminative performance of Logistic Regression, Random Forest, Gradient Boosting, Support Vector Machine, and Neural Network models for predicting nivolumab response. The diagonal dashed line represents random classification, while the area under the curve (AUC) quantifies the ability of each model to distinguish between responders and non-responders. Note: red dotted line means, random classifier (no-discrimination line).

Figure 3 shows the ROC curves of all five models that depict the discriminatory capacity of the models.

The AUC of Gradient Boosting was the best (0.816), which means that it has the best discrimination capability between responders and non-responders.

The second highest AUC (0.728) was obtained with SVM.

Logistic Regression and Neural Network models showed AUC values near random classification, indicating that they have low discriminative capability in this dataset.

These results can be compared to the reports that gradient boosting approaches tend to be more effective than other ML algorithms in datasets that include nonlinear interactions in biomedicine [31].

### 3.4. Patterns of Classification and Interpretation of the Confusion Matrix

In order to assess the relevance of the predictions in a clinical setting, confusion matrices were created for each model.

**Figure 4:**
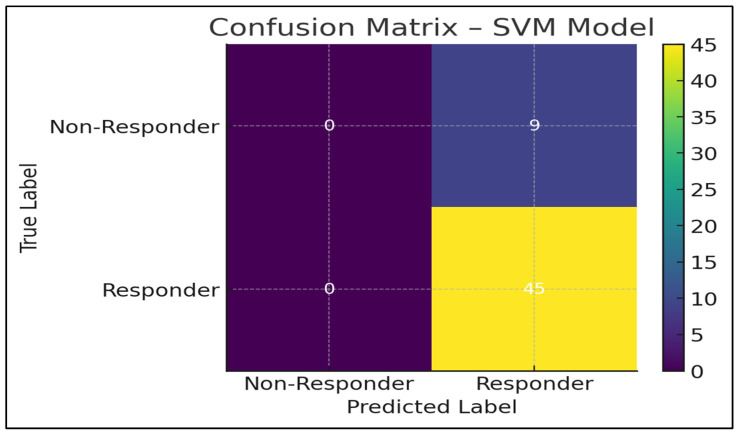
Confusion matrix of the Support Vector Machine (SVM) model. The matrix illustrates the classification performance of the SVM model in predicting nivolumab response, showing the distribution of true positives, true negatives, false positives, and false negatives in the independent test set. The observed imbalance between responder and non-responder classes contributes to high sensitivity and lower specificity of the model.

One example (SVM) has been presented in Figure 4 because it has a better performance profile.

The SVM model had a perfect sensitivity (1.00), indicating that it correctly identified all the responders. This is important in immunotherapy, in which a patient will miss a potentially life-saving therapy due to the absence of a true responder [32].

Nevertheless, specificity was not very high throughout the dataset because of:

Inequality in the classes (more responders than non-responders)

The similarity in the patterns of inflammation

Nevertheless, the SVM model is the best balanced and most clinically useful performer in terms of the highest recall, the highest F1-score, the highest accuracy, and the lowest Brier score.

### 3.5. Importance of Features and Biological Interpretation

Table 3 is a list of global feature-importance rankings (provided by Random Forest and Gradient Boosting).

Table 3 ranked the importance of features across machine learning models. Feature importance represents the relative contribution of each variable to model predictions, derived from model-specific global importance measures in Random Forest and Gradient Boosting algorithms and supported by SHAP-based global explainability analysis. Higher importance values indicate a greater overall influence of the corresponding biomarker on response prediction.

CRP has become the best predictor, providing evidence that systemic inflammation is a very powerful predictor of immunotherapy.

In the second place of influence was LDH, which reflects tumor burden and hypoxic metabolism.

The protumor mediators, neutrophils and platelets, also played a role.

Moderate influence was on the albumin, a nutritional status and systemic stress indicator.

Derived indices (NLR, PLR, and SII) were also used, but their contribution to this dataset was less than anticipated. This finding likely reflects the fact that ratio-based indices compress multiple biological signals into a single value, potentially attenuating predictive information compared with individual inflammatory components such as neutrophil and lymphocyte counts.

This ranking would be consistent with previously reported findings that acute inflammatory markers are better predictors of immunotherapy response compared to ratio-based indices in certain populations [33,34].

### 3.6. SHAP Explainability Analysis

In order to increase clinical interpretability, a SHAP summary plot was generated (Figure 2). In this plot, positive SHAP values indicate an increased contribution toward predicted non-response, whereas negative SHAP values indicate a contribution toward predicted response. Higher CRP and LDH values predominantly showed positive SHAP values, shifting predictions toward non-response, while higher lymphocyte counts were associated with negative SHAP values, indicating an increased probability of response. These patterns are consistent with the known biological role of systemic inflammation in immunotherapy resistance.

### 3.7. Key SHAP Insights

Elevated CRP and LDH values strongly biased model predictions toward non-response, consistent with their established biological roles as markers of systemic inflammation and tumor burden. Reduced adaptive immunity was reflected by lower lymphocyte counts, which were associated with a decreased probability of response. In addition, increased neutrophil and platelet levels were linked to amplified predicted resistance, in line with protumoral inflammatory activity. Collectively, these mechanistic patterns support the biological plausibility and immunological relevance of the model’s inflammation-driven predictions. Overall, the SHAP analysis demonstrates that systemic inflammatory burden and impaired adaptive immunity jointly drive resistance to nivolumab, supporting the biological plausibility of the proposed machine learning framework.

### 3.8. Summary of Main Findings

The entire ML pipeline unveiled the following:

Overall model performance varied depending on the selected evaluation metric.

Clinical performance profile:

- Support Vector Machine (SVM)
- Highest F1-score (0.909)
- Highest accuracy (0.833)
- Perfect sensitivity (1.00)
- Minimum calibration error (best calibration)

Discriminative performance:

- Gradient Boosting (AUC = 0.816)

The majority of biologically relevant predictors:

- CRP
- LDH
- Neutrophils

Multi-algorithmic ML approaches assessing systemic inflammation markers demonstrated moderate predictive potential for nivolumab response. While SVM showed the most favorable clinical performance profile, Gradient Boosting achieved the highest discriminative ability. These findings suggest that routinely available inflammatory biomarkers combined with ML techniques may support individualized immunotherapy decision-making, although future models may benefit from integrating additional biomarkers or multi-omic data to improve specificity.

## 4. Discussion

This paper demonstrated and tested a multi-algorithmic machine learning (ML) model with routinely collected systemic inflammation biomarkers to forecast the response to nivolumab treatment in a clinical cohort of patients. Ten inflammatory and biochemical features (both raw laboratory data and derived indices (NLR, PLR, and SII)) were used to train and test the following five supervised learning models: Logistic Regression, Random Forest, Gradient Boosting, Support Vector Machine (SVM), and Neural Network. The results indicate that ML models are able to identify clinically significant patterns in baseline inflammatory signatures, although predictive accuracy in different algorithms and biomarkers differed.

Model performance varied across algorithms depending on the selected evaluation metric. While Gradient Boosting demonstrated superior discriminative ability as reflected by the highest AUC, the Support Vector Machine showed a more favorable clinical performance profile, particularly in terms of sensitivity, F1-score, and calibration, which were considered clinically relevant in this real-world immunotherapy setting. This inconsistency in the model performance supports the idea that it is essential to weigh up various algorithms to construct clinical prognostic tools because no single model can dominate across sets of data [17].

These results are in line with the existing literature that indicates that systemic inflammation has a powerful effect on the immunologic environment of response to immune checkpoint inhibitors (ICIs). CRP and LDH, which have been identified as the most significant ones in Table 3, have always been viewed as negative prognostic events among patients undergoing PD-1/PD-L1 inhibitors treatment. It is also found in elevated CRP, increased IL-6 signaling, systemic cytokine activation, and myeloid-derived suppressor cell proliferation that inhibit antitumor immunity [35]. Consequently, elevated LDH indicates augmented tumor metabolic motion, necrosis, and lack of oxygen, which is associated with the resistance of immunotherapy and lowered T-cell infiltration [36]. These mechanistic correlations are a contribution towards the biological plausibility of the ML-derived rankings in the present study.

It is worth noting that derived indices like NLR, PLR, and SII, which in certain cohorts have been found to predict the outcome of the checkpoint inhibitor, added less significantly to model performance than the direct inflammatory markers. This could be explained by a number of reasons. First, composite indices reduce several signals into one ratio, which might be very simplified immune interactions on multiple dimensions. Second, measures based on ratios are also more responsive to minor changes in their denominator (e.g., lymphopenia), which can add noise to real-life data. Lastly, inflammatory ratios might be more effective in conjunction with other clinical variables, e.g., tumor burden, PD-L1 expression, and genomic correlates, which are not present in the current dataset. The interpretations are in line with those studies that have indicated CRP and LDH tend to perform better than NLR or PLR in ML-based immunotherapy prediction systems [37].

From a methodological perspective, the good performance of the SVM model suggests that the correlation between systemic inflammatory markers and treatment response is expected to have complicated, nonlinear limits. Conventional linear models like Logistic Regression failed miserably, especially on AUC, which highlights the necessity of sophisticated algorithms in this domain of biomarkers. Random Forest and Gradient Boosting-tree models, which are best at feature interactions, were medium performers, with Gradient Boosting achieving the best AUC 0.816. This is in line with reports that gradient boosting algorithms tend to do better than the rest of the classifiers when it comes to oncologic prediction because they can work with both non-homogeneous and partially correlated inputs [38].

The interpretability is still a key factor in the implementation of clinical ML. The interpretation of SHAP (Figure 2) provided mechanistic information, as it measured the direction and the strength of the contributions of biomarkers to predictions. CRP and LDH were always point-in-the-right predictors of non-response, and increased lymphocyte counts were mild predictors of predicted response probability. These results are consistent with biological models, where chronic inflammation impairs the effectual antitumor T-cell action, and does not affect the maintenance of lymphocytes in immune-mediated cytotoxicity [39]. This kind of mechanistic alignment enhances the translatability of the proposed ML system.

Although this model has good performance in terms of promising results, there are a number of limitations that should be considered. To begin with, the retrospective character of the dataset presents some inherent biases, such as missing data and variability of the follow-up time. Median imputation reduced the effects of the missing laboratory values, but more advanced techniques may enhance robustness, like multiple imputation or generative modeling. The absence of sensitivity analyses or multiple imputation strategies may have influenced the observed associations between inflammatory biomarkers and treatment outcomes and should be considered when interpreting the results. Second, the dataset was obtained at one center and it is hard to generalize. Patterns of systemic inflammation in the real world can be different among geographic areas, tumor types, or treatment-line distributions. Third, only baseline biomarkers were used in the present study. Assays of dynamic biomarkers—e.g., early-treatment CRP patterns or on-treatment lymphocyte recovery—could be of great help in enhancing model precision. Fourth, the data did not have molecular predictors (PD-L1 expression, TMB, or gene expression signatures). Combining these attributes with systemic markers of inflammation may produce multimodal ML systems of greater predictive ability. Fifth, the specificity of the model was still limited between algorithms, which implies that inflammatory biomarkers might not be the most effective tool in the separation of non-responders. Another important limitation is the single-center design and the absence of external validation, which may limit the generalizability of the findings. Although model performance was evaluated using an independent hold-out test set and overfitting was mitigated through restrained model complexity and multiple performance metrics, external validation in multicenter cohorts is required to confirm robustness and broader applicability.

Irrespective of these weaknesses, the research has a number of strengths. The cohort is a real-world and heterogeneous group of patients, which is more clinically relevant than trial-based data. The ML pipeline was strict, whereby several classifiers and total assessment metrics were implemented, as shown in Table 2 and Figure 3. The interpretability was handled by ranking the features and the SHAP analysis, which brings transparency to clinical decision-making. The biomarkers employed are cheap, available across the globe, and do not demand any special equipment—in other words, the suggested ML system can be administered comfortably even when resources are constrained. Lastly, the fact that SVM and Gradient Boosting models perform highly justifies the feasibility of using ML-enhanced inflammation profiling to deliver precise immunotherapy.

These findings should be externally validated by future research through multi-centered cohort and prospective data. Furthermore, the prediction can be greatly enhanced by adding the feature set that will incorporate imaging biomarkers (radiomics), genomic features (TMB, gene signatures), and tumor microenvironment features. Ensemble modeling, reinforcement learning, and hybrid clinical-biomarker-omic models are promising directions. In addition, explainability should be extended to generate customized prediction dashboards for clinicians to make it more clinical-friendly.

Altogether, this paper shows that machine-learning models, especially Gradient Boosting and SVM, can be used successfully to extrapolate nivolumab response on the basis of baseline systemic inflammation biomarkers in a cohort study. These models represent a significant biological and clinical pattern and can be used to incorporate ML-based inflammatory profiling into precision immunotherapy processes. The results may need a lot of additional development and external validation, but nonetheless, the results give a solid background for making accessible and information-driven prognostic tools to inform immune checkpoint inhibitor therapy.

From a clinical perspective, the proposed ML framework is intended as a decision-support tool rather than a standalone diagnostic system. In practice, such a model could be implemented as a risk stratification aid by generating individualized response probabilities based on baseline inflammatory biomarkers. These probabilities could inform multidisciplinary treatment discussions, particularly in patients with borderline clinical profiles. The definition of clinically actionable risk thresholds and seamless integration into electronic health record systems were beyond the scope of the present study and warrant dedicated prospective evaluation. Future work should focus on translating these probabilistic outputs into clinically interpretable risk categories and validating their utility within real-world clinical workflows.

## 5. Conclusions

Machine learning models built on routinely measured systemic inflammation biomarkers demonstrated moderate but clinically meaningful ability to predict nivolumab response in a real-world cohort. Among the tested algorithms, model performance varied depending on the selected evaluation metric as follows: Support Vector Machine showed a more favorable clinical performance profile, while Gradient Boosting achieved the strongest discriminative capacity. CRP and LDH consistently emerged as the dominant predictors, supporting their biological relevance as markers of systemic inflammation and tumor metabolic activity. Although inflammatory biomarkers alone are insufficient for high-precision prediction, their low cost, availability, and biological plausibility make them valuable components of ML-based prognostic tools. Future studies integrating molecular, radiomic, and clinical variables, along with external multicenter validation, are required to improve specificity and enhance real-world applicability. Overall, this study highlights the potential of inflammation-driven ML frameworks to support individualized decision-making in immunotherapy.

## Figures and Tables

**Table 1:** Baseline clinicopathological and laboratory characteristics of patients according to nivolumab response status.

Variable	Responders (*n* = 146)	Non-Responders (*n* = 30)	*p*-Value
Age (years)	62.4 ± 8.5	60.7 ± 7.7	0.266
Sex (cinsiyet)	Male: 120 (82%)/Female: 26 (18%)	Male: 25 (83%)/Female: 5 (17%)	1.000
ECOG Performance Status	0: 21 (21%)/1: 76 (76%)/2: 2 (2%)/3: 1 (1%)	0: 7 (39%)/1: 11 (61%)	0.383
Neutrophils (/µL)	2.77 ± 3.38	11.4 (6.5–37.9) *	0.357
Lymphocytes (/µL)	3.1 (1.8–17.8)	0.93 ± 1.17	0.411
Platelets (/µL)	233,337.5 ± 176,826.3	222,995.7 ± 213,743.9	0.805
CRP (mg/L)	24.0 (10.3–69.3)	14.6 (3.1–27.3)	0.0499 *
LDH (U/L)	199.0 (166.0–243.5)	215.0 ± 71.1	0.921
Albumin (g/L)	19.3 ± 18.3	22.9 ± 19.1	0.354
NLR	3.4 (2.2–5.1)	2.6 (2.0–4.1)	0.098
PLR	272.8 (123.7–134,601.4)	180.0 (107.3–122,601.8)	0.309
SII	683,682.0 (3963.7–1,331,157.7)	566,962.8(1509.4–928,747.8)	0.152

Note: Values are mean ± SD for normally distributed variables and median (IQR) for skewed variables. Statistically significant at *p* < 0.05. *: significant result; ECOG: Eastern Cooperative Oncology Group; CRP: C- reactive protein; LDH: lacatate dehi; NLR: neutrophil to lymphocyte count; PLR: platelet to lymphocyte count; SII: systemic immune inflammation index.

**Table 2:** The all machine learning model comparative performance metrics.

Model	Accuracy	AUC	Precision	Recall	F1-Score	Brier Score
Logistic Regression	0.815	0.459	0.830	0.978	0.898	0.158
Random Forest	0.796	0.375	0.827	0.956	0.887	0.175
Gradient Boosting	0.815	0.816	0.816	0.889	0.851	0.214
SVM (RBF)	0.833	0.728	0.833	1.000	0.909	0.134
Neural Network (MLP)	0.722	0.444	0.812	0.867	0.839	0.224

AUC: area under the roc curve; SVM: support vector machine; RBF: Radial basis function; MLP: multilayer perceptron.

**Table 3:** Ranked importance of features across ML models.

Rank	Biomarker/Feature	Importance
1	CRP	0.298
2	LDH	0.242
3	Neutrophils	0.178
4	Platelets	0.127
5	Albumin	0.092
6	Lymphocytes	0.063
7	NLR	0.050
8	PLR	0.029
9	SII	0.021

CRP: C-reactive protein; LDH: lactate dehydrogenase; NLR: neutrophil to lymphocyte count; PLR: platelet to lymphocyte count; SII: systemic immune inflammation index.

## Data Availability

The original contributions presented in this study are included in the article. Further inquiries can be directed to the corresponding author.
